# Crystal structure of (*E*)-1,3-bis­(6-methoxy­naphthalen-2-yl)prop-2-en-1-one

**DOI:** 10.1107/S2056989015019714

**Published:** 2015-10-24

**Authors:** Paresh N. Patel, Anju Chadha

**Affiliations:** a Department of Biotechnology, Indian Institute of Technology Madras, Chennai 600 036, India; b Department of Biotechnology and National Center for Catalysis Research, Indian Institute of Technology Madras, Chennai 600 036, India

**Keywords:** crystal structure, bis-naphthalene, chalcone, C—H⋯π inter­actions

## Abstract

In the title compound, C_25_H_20_O_3_, the central –C(=O)—C=C– chain is disordered over two positions about the central C atom, with an occupancy ratio of 0.848 (6):0.152 (6). The mol­ecule is twisted with the two naphthalene ring systems being inclined to one another by 52.91 (9)°. In the crystal, mol­ecules are linked by C—H⋯π inter­actions, forming a three-dimensional structure. The structure was refined as a two-component twin with a 180 ° rotation about the c* axis.

## Related literature   

For natural sources of chalcones and their derivatives, see: Anderson & Markham (2006 ▸); Yadav *et al.* (2011 ▸). For examples of their biological activities, see: Liu *et al.* (2011 ▸); Siddiqui *et al.* (2012 ▸). For their use as synthons for the preparation of five- and six-membered ring systems, see: Powers *et al.* (1998 ▸). For their use as inter­mediates in the synthesis of pharmaceutical mol­ecules, see: Perozo-Rondon *et al.* (2006 ▸). For the crystal structure of a closely related compound, 3-(6-meth­oxy-2-naphth­yl)-1-(2-naphth­yl)prop-2-en-1-one, see: Yathirajan *et al.* (2006 ▸).
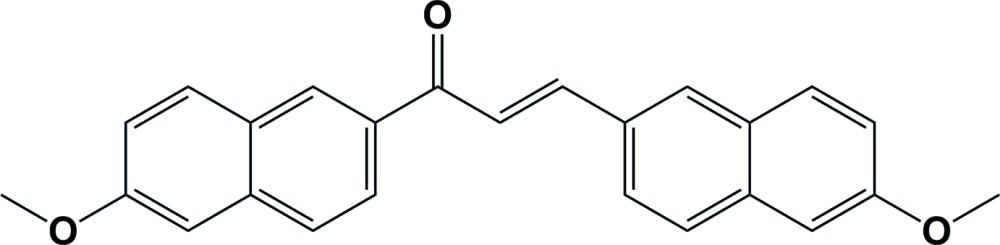


## Experimental   

### Crystal data  

C_25_H_20_O_3_
*M* *_r_* = 368.41
Monoclinic 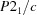

*a* = 6.027 (5) Å
*b* = 19.926 (5) Å
*c* = 15.415 (5) Å
β = 90.366 (5)°
*V* = 1851.2 (17) Å^3^
*Z* = 4
Mo *K*α radiation
μ = 0.09 mm^−1^
*T* = 293 K
0.30 × 0.20 × 0.20 mm

### Data collection  

Bruker Kappa APEXII CCD diffractometer
Absorption correction: multi-scan (*SADABS*: Bruker, 2004 ▸) *T*_min_ = 0.932, *T*_max_ = 0.951
3345 measured reflections
3345 independent reflections
1837 reflections with *I* > 2σ(*I*)
*R*_int_ = 0.072

### Refinement  

*R*[*F* ^2^ > 2σ(*F* ^2^)] = 0.061
*wR*(*F* ^2^) = 0.179
*S* = 1.13
3345 reflections
267 parameters
2 restraints
H-atom parameters constrained
Δρ_max_ = 0.19 e Å^−3^
Δρ_min_ = −0.16 e Å^−3^

### 

Data collection: *APEX2* (Bruker, 2004 ▸); cell refinement: *APEX2* and *SAINT* (Bruker, 2004 ▸); data reduction: *SAINT* and *XPREP* (Bruker, 2004 ▸); program(s) used to solve structure: *SIR92* (Altomare *et al.*, 1993 ▸); program(s) used to refine structure: *SHELXL2014* (Sheldrick, 2015 ▸); molecular graphics: *ORTEP-3 for Windows* (Farrugia, 2012 ▸) and *Mercury* (Macrae *et al.*, 2008 ▸); software used to prepare material for publication: *SHELXL2014* and *PLATON* (Spek, 2009 ▸).

## Supplementary Material

Crystal structure: contains datablock(s) global, I. DOI: 10.1107/S2056989015019714/su5219sup1.cif

Structure factors: contains datablock(s) I. DOI: 10.1107/S2056989015019714/su5219Isup2.hkl

Supporting information file. DOI: 10.1107/S2056989015019714/su5219Isup3.cml

**Fig. 1.**
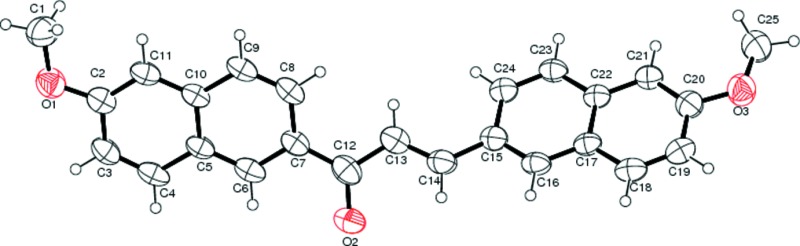
The molecular structure of the title compound, with atom labelling. Displacement ellipsoids are drawn at the 50% probability level. Only the major component of the disordered O atom is shown.

**Fig. 2.**
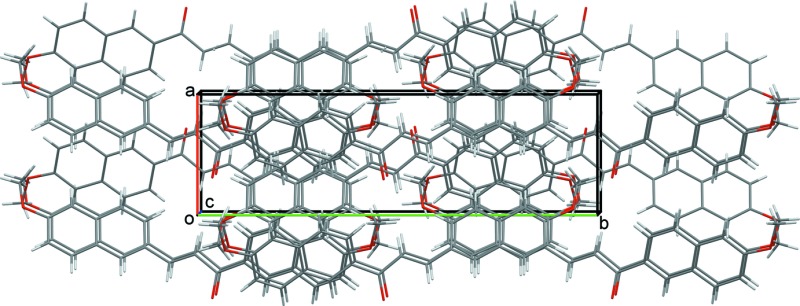
A view along the c axis of the crystal packing of the title compound.

. DOI: 10.1107/S2056989015019714/su5219fig1.tif
The mol­ecular structure of the title compound, with atom labelling. Displacement ellipsoids are drawn at the 50% probability level. Only the major component of the disordered O atom is shown.

c . DOI: 10.1107/S2056989015019714/su5219fig2.tif
A view along the *c* axis of the crystal packing of the title compound.

CCDC reference: 1432044

Additional supporting information:  crystallographic information; 3D view; checkCIF report

## Figures and Tables

**Table 1:** Hydrogen-bond geometry (, ) *Cg*2 and *Cg*4 are the centroids of rings C5-C10 and C17-C22, respectively.

*D*H*A*	*D*H	H*A*	*D* *A*	*D*H*A*
C9H9*Cg*4^i^	0.93	2.86	3.543(4)	131
C18H18*Cg*2^ii^	0.93	2.85	3.611(4)	140
C23H23*Cg*2^iii^	0.93	2.88	3.592(4)	134

Symmetry codes: (i) 
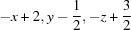
; (ii) 
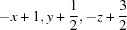
; (iii) 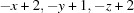.

## supplementary crystallographic information

### S1. Comments

Hetero­aryl chalcones are well documented as important synthons for a number of pharmaceutically active molecules, and extensive investigations have demonstrated the biological properties of natural and synthetic chalcones. These properties are largely attributed to the presence of the a,ß-unsaturated ketone moiety in the chalcone (Anderson & Markham, 2006; Yadav *et al.*, 2011). Chalcones are reported to possess many useful properties, for example anti­bacterial [Liu *et al.*, 2011] and anti­fungal [Siddiqui *et al.*, 2012]. These compounds are important synthons for the preparation of five- and six-membered ring systems [Powers *et al.*, 1998] as well as inter­mediates in the synthesis of many pharmaceutically useful molecules [Perozo-Rondon *et al.*, 2006]. Given such varied pharmacological activities and synthetic utilities, chalcones have always attracted chemists to develop newer molecules and study their biological activities. Adding to the list of active hetero­aryl chalcones for use in pharmaceutical applications and as an effective synthon for the preparation of five- and six-member ring systems, we report herein on the synthesis and crystal structure of the title compound.

In the title compound, Fig. 1, the central -C12(═O2)—C13═C14- chain is disordered over two positions about the central atom C13 with an occupancy ratio of 0.848 (6):0.152 (6) for atom O2 (O2A:O2B). The molecule is twisted with the two naphthalene ring systems being inclined to one another by 52.91 (9)°. This situation is similar to that in compound 3-(6-meth­oxy-2-naphthyl)-1-(2-naphthyl)­prop- 2-en-1-one (Yathirajan *et al.*, 2006), where the two naphthalene ring systems are inclined to one another by 54.41 (3) °.

In the crystal, molecules are linked by C—H···π inter­actions forming a three-dimensional structure. There are no other intra- or inter-molecular inter­actions present.

### S2. Synthesis and crystallization

To a stirred solution of 6-meth­oxy-2-naphthaldehyde (1.86 g, 10 mmol) in ethanol (10 ml), 1-(6-meth­oxy­naphthalen-2-yl) ethanone (2.00 g, 10 mmol) dissolved in ethanol (10 ml) was added portion wise. The reaction mixture was stirred at room temperature for an additional 20 min, during which time it turned to a homogeneous solution. KOH solution (40%, 2 ml) was then added drop wise and the resultant mixture was stirred at room temperature for 2 h. The precipitated product was then collected by filtration and purified by recrystallization from chloro­form–methanol (1:1 v/v, 10 ml), to afford 2.29 g (82%) of the title compound as yellow–brown needles (m.p.: 396-397 K). Colourless block-like crystals, suitable for X-ray diffraction, were obtained by crystallization from a 1 ml saturated solution in ethanol.

### S3. Refinement

Crystal data, data collection and structure refinement details are summarized in Table 2. The H atoms were included in calculated positions and treated as riding atoms: C—H = 0.93-0.96 Å with U_iso_(H) = 15.U_eq_(C-methyl) and 1.2U_eq_(C) for other H atoms. The central -C12(═O2)—C13═C14- chain is disordered over two positions about the central atom C13 with an occupancy ratio of 0.848 (6):0.152 (6) for atom O2 (O2A:O2B). The structure was refined as a two-component twin: 180 ° rotation about the c* axis; BASF = 0.063 (1).

### Crystal data

**Table:**

C_25_H_20_O_3_	*F*(000) = 776
*M**_r_* = 368.41	*D*_x_ = 1.322 Mg m^−^^3^
Monoclinic, *P*2_1_/*c*	Mo *K*α radiation, λ = 0.71073 Å
*a* = 6.027 (5) Å	Cell parameters from 4691 reflections
*b* = 19.926 (5) Å	θ = 2.6–22.4°
*c* = 15.415 (5) Å	µ = 0.09 mm^−^^1^
β = 90.366 (5)°	*T* = 293 K
*V* = 1851.2 (17) Å^3^	Block, colourless
*Z* = 4	0.30 × 0.20 × 0.20 mm

### Data collection

**Table:**

Bruker Kappa APEXII CCD diffractometer	1837 reflections with *I* > 2σ(*I*)
Radiation source: Sealed tube	*R*_int_ = 0.072
ω and φ scan	θ_max_ = 25.3°, θ_min_ = 2.4°
Absorption correction: multi-scan (*SADABS*: Bruker, 2004)	*h* = −7→7
*T*_min_ = 0.932, *T*_max_ = 0.951	*k* = −23→23
3345 measured reflections	*l* = 0→18
3345 independent reflections	

### Refinement

**Table:**

Refinement on *F*^2^	Hydrogen site location: inferred from neighbouring sites
Least-squares matrix: full	H-atom parameters constrained
*R*[*F*^2^ > 2σ(*F*^2^)] = 0.061	*w* = 1/[σ^2^(*F*_o_^2^) + (0.076*P*)^2^ + 0.242*P*] where *P* = (*F*_o_^2^ + 2*F*_c_^2^)/3
*wR*(*F*^2^) = 0.179	(Δ/σ)_max_ < 0.001
*S* = 1.13	Δρ_max_ = 0.19 e Å^−^^3^
3345 reflections	Δρ_min_ = −0.16 e Å^−^^3^
267 parameters	Extinction correction: *SHELXL2014* (Sheldrick, 2015), Fc^*^=kFc[1+0.001xFc^2^λ^3^/sin(2θ)]^-1/4^
2 restraints	Extinction coefficient: 0.0042 (12)

### Special details

**Table:**

Geometry. All e.s.d.'s (except the e.s.d. in the dihedral angle between two l.s. planes) are estimated using the full covariance matrix. The cell e.s.d.'s are taken into account individually in the estimation of e.s.d.'s in distances, angles and torsion angles; correlations between e.s.d.'s in cell parameters are only used when they are defined by crystal symmetry. An approximate (isotropic) treatment of cell e.s.d.'s is used for estimating e.s.d.'s involving l.s. planes.
Refinement. Refined as a 2-component twin.

### Fractional atomic coordinates and isotropic or equivalent isotropic displacement parameters (Å^2^)

**Table:**

	*x*	*y*	*z*	*U*_iso_*/*U*_eq_	Occ. (<1)
O1	0.7149 (4)	0.07041 (12)	0.81151 (15)	0.0752 (7)	
O2A	0.3022 (4)	0.46629 (14)	0.8854 (2)	0.0831 (12)	0.848 (6)
O2B	0.3818 (18)	0.5689 (8)	0.9159 (10)	0.075 (6)	0.152 (6)
O3	1.0538 (4)	0.94015 (12)	0.91441 (16)	0.0810 (8)	
C1	0.9200 (6)	0.05490 (18)	0.7708 (3)	0.0878 (12)	
H1A	0.9265	0.0769	0.7155	0.132*	
H1B	0.9311	0.0072	0.7628	0.132*	
H1C	1.0405	0.0701	0.8068	0.132*	
C2	0.6696 (5)	0.13625 (18)	0.82763 (19)	0.0588 (9)	
C3	0.4657 (5)	0.14718 (18)	0.8680 (2)	0.0637 (9)	
H3	0.3764	0.1108	0.8821	0.076*	
C4	0.3971 (5)	0.20984 (18)	0.88679 (19)	0.0624 (9)	
H4	0.2601	0.2161	0.9130	0.075*	
C5	0.5308 (4)	0.26629 (16)	0.86723 (18)	0.0507 (8)	
C6	0.4623 (5)	0.33174 (17)	0.88371 (18)	0.0582 (9)	
H6	0.3225	0.3389	0.9071	0.070*	
C7	0.5958 (5)	0.38617 (16)	0.86635 (19)	0.0568 (8)	
C8	0.8060 (5)	0.37394 (18)	0.8290 (2)	0.0631 (9)	
H8	0.9001	0.4098	0.8174	0.076*	
C9	0.8711 (5)	0.31106 (18)	0.8100 (2)	0.0619 (9)	
H9	1.0084	0.3045	0.7842	0.074*	
C10	0.7380 (4)	0.25513 (16)	0.82814 (18)	0.0513 (8)	
C11	0.8029 (5)	0.18913 (17)	0.80906 (19)	0.0576 (8)	
H11	0.9397	0.1815	0.7833	0.069*	
C12	0.5122 (6)	0.45453 (18)	0.8827 (2)	0.0683 (10)	
H12B	0.3602	0.4619	0.8864	0.082*	0.152 (6)
C13	0.6636 (5)	0.51023 (18)	0.8930 (2)	0.0664 (9)	
H13A	0.8128	0.5016	0.9039	0.080*	
C14	0.5946 (5)	0.57381 (18)	0.8872 (2)	0.0660 (9)	
H14A	0.4446	0.5796	0.8747	0.079*	0.848 (6)
C15	0.7225 (5)	0.63517 (17)	0.89786 (19)	0.0548 (8)	
C16	0.6339 (5)	0.69511 (17)	0.87011 (19)	0.0575 (9)	
H16	0.4948	0.6948	0.8436	0.069*	
C17	0.7436 (4)	0.75620 (16)	0.87992 (18)	0.0514 (8)	
C18	0.6536 (5)	0.81769 (19)	0.8512 (2)	0.0628 (9)	
H18	0.5163	0.8177	0.8234	0.075*	
C19	0.7603 (5)	0.87635 (18)	0.8629 (2)	0.0656 (9)	
H19	0.6986	0.9161	0.8423	0.079*	
C20	0.9658 (5)	0.87709 (17)	0.9066 (2)	0.0613 (9)	
C21	1.0620 (5)	0.81927 (16)	0.93433 (18)	0.0534 (8)	
H21	1.1999	0.8206	0.9617	0.064*	
C22	0.9538 (4)	0.75732 (16)	0.92185 (17)	0.0491 (7)	
C23	1.0433 (5)	0.69571 (16)	0.94924 (19)	0.0536 (8)	
H23	1.1821	0.6953	0.9759	0.064*	
C24	0.9338 (5)	0.63677 (16)	0.93804 (19)	0.0573 (8)	
H24	0.9986	0.5970	0.9570	0.069*	
C25	1.2506 (6)	0.94859 (18)	0.9618 (3)	0.0897 (12)	
H25A	1.2274	0.9351	1.0209	0.135*	
H25B	1.2941	0.9949	0.9603	0.135*	
H25C	1.3654	0.9215	0.9368	0.135*	

### Atomic displacement parameters (Å^2^)

**Table:**

	*U*^11^	*U*^22^	*U*^33^	*U*^12^	*U*^13^	*U*^23^
O1	0.0769 (16)	0.0723 (18)	0.0765 (16)	−0.0081 (12)	0.0102 (13)	0.0067 (13)
O2A	0.0531 (19)	0.084 (2)	0.112 (2)	−0.0078 (14)	0.0012 (15)	0.0059 (17)
O2B	0.039 (9)	0.096 (13)	0.091 (12)	0.008 (7)	−0.008 (7)	−0.015 (9)
O3	0.0854 (17)	0.0663 (17)	0.0910 (18)	−0.0005 (13)	−0.0171 (14)	0.0050 (13)
C1	0.081 (3)	0.089 (3)	0.093 (3)	0.006 (2)	0.014 (2)	0.006 (2)
C2	0.055 (2)	0.071 (2)	0.0501 (19)	−0.0080 (17)	−0.0043 (15)	0.0090 (17)
C3	0.057 (2)	0.073 (3)	0.061 (2)	−0.0186 (18)	0.0040 (16)	0.0117 (19)
C4	0.0439 (17)	0.092 (3)	0.052 (2)	−0.0166 (18)	0.0061 (14)	0.0073 (19)
C5	0.0416 (16)	0.071 (2)	0.0393 (16)	−0.0106 (15)	−0.0027 (13)	0.0049 (16)
C6	0.0461 (18)	0.088 (3)	0.0403 (17)	−0.0165 (17)	−0.0003 (13)	−0.0003 (17)
C7	0.0517 (19)	0.069 (2)	0.0495 (19)	−0.0129 (16)	−0.0015 (14)	−0.0002 (16)
C8	0.056 (2)	0.072 (3)	0.061 (2)	−0.0228 (17)	0.0017 (16)	0.0061 (18)
C9	0.0464 (18)	0.084 (3)	0.056 (2)	−0.0174 (17)	0.0040 (15)	0.0064 (19)
C10	0.0419 (16)	0.072 (2)	0.0400 (16)	−0.0126 (16)	−0.0033 (13)	0.0063 (16)
C11	0.0439 (17)	0.079 (2)	0.0501 (19)	−0.0083 (17)	0.0025 (14)	0.0114 (17)
C12	0.070 (2)	0.082 (3)	0.053 (2)	−0.021 (2)	0.0053 (17)	0.0013 (18)
C13	0.058 (2)	0.075 (3)	0.066 (2)	−0.0085 (19)	−0.0023 (16)	0.0023 (19)
C14	0.0477 (19)	0.082 (3)	0.069 (2)	−0.0019 (18)	−0.0021 (16)	−0.014 (2)
C15	0.0490 (18)	0.068 (2)	0.0475 (18)	0.0004 (16)	−0.0006 (14)	−0.0124 (16)
C16	0.0439 (17)	0.077 (3)	0.0512 (19)	0.0023 (16)	−0.0057 (14)	−0.0156 (17)
C17	0.0464 (17)	0.065 (2)	0.0428 (17)	0.0082 (16)	−0.0002 (14)	−0.0077 (15)
C18	0.0486 (18)	0.086 (3)	0.053 (2)	0.0098 (18)	−0.0033 (15)	−0.0082 (19)
C19	0.063 (2)	0.071 (3)	0.063 (2)	0.0156 (18)	−0.0007 (17)	0.0020 (18)
C20	0.063 (2)	0.064 (2)	0.057 (2)	0.0004 (18)	0.0026 (16)	−0.0042 (17)
C21	0.0472 (17)	0.066 (2)	0.0469 (18)	0.0018 (16)	−0.0007 (14)	0.0005 (16)
C22	0.0457 (16)	0.063 (2)	0.0389 (16)	0.0029 (15)	0.0028 (13)	−0.0048 (15)
C23	0.0441 (17)	0.070 (2)	0.0469 (18)	0.0016 (16)	−0.0069 (13)	−0.0036 (16)
C24	0.0536 (19)	0.066 (2)	0.0521 (19)	0.0056 (16)	−0.0011 (15)	−0.0022 (16)
C25	0.081 (3)	0.076 (3)	0.112 (3)	−0.006 (2)	−0.013 (2)	0.000 (2)

### Geometric parameters (Å, º)

**Table:**

O1—C2	1.363 (4)	C12—C13	1.445 (4)
O1—C1	1.423 (4)	C12—H12B	0.9300
O2A—C12	1.288 (4)	C13—C14	1.336 (4)
O2B—C14	1.363 (11)	C13—H13A	0.9300
O3—C20	1.369 (4)	C14—C15	1.454 (4)
O3—C25	1.400 (4)	C14—H14A	0.9300
C1—H1A	0.9600	C15—C16	1.375 (4)
C1—H1B	0.9600	C15—C24	1.413 (4)
C1—H1C	0.9600	C16—C17	1.393 (4)
C2—C11	1.357 (4)	C16—H16	0.9300
C2—C3	1.398 (4)	C17—C18	1.410 (4)
C3—C4	1.347 (4)	C17—C22	1.419 (4)
C3—H3	0.9300	C18—C19	1.346 (4)
C4—C5	1.417 (4)	C18—H18	0.9300
C4—H4	0.9300	C19—C20	1.406 (5)
C5—C6	1.392 (4)	C19—H19	0.9300
C5—C10	1.408 (4)	C20—C21	1.358 (4)
C6—C7	1.377 (4)	C21—C22	1.409 (4)
C6—H6	0.9300	C21—H21	0.9300
C7—C8	1.416 (4)	C22—C23	1.405 (4)
C7—C12	1.475 (4)	C23—C24	1.358 (4)
C8—C9	1.346 (4)	C23—H23	0.9300
C8—H8	0.9300	C24—H24	0.9300
C9—C10	1.402 (4)	C25—H25A	0.9600
C9—H9	0.9300	C25—H25B	0.9600
C10—C11	1.404 (4)	C25—H25C	0.9600
C11—H11	0.9300		
			
C2—O1—C1	117.7 (3)	C14—C13—H13A	119.2
C20—O3—C25	118.9 (3)	C12—C13—H13A	119.2
O1—C1—H1A	109.5	C13—C14—O2B	101.7 (7)
O1—C1—H1B	109.5	C13—C14—C15	128.7 (3)
H1A—C1—H1B	109.5	O2B—C14—C15	121.5 (7)
O1—C1—H1C	109.5	C13—C14—H14A	115.6
H1A—C1—H1C	109.5	C15—C14—H14A	115.6
H1B—C1—H1C	109.5	C16—C15—C24	117.6 (3)
C11—C2—O1	126.1 (3)	C16—C15—C14	119.4 (3)
C11—C2—C3	119.8 (3)	C24—C15—C14	123.0 (3)
O1—C2—C3	114.1 (3)	C15—C16—C17	122.8 (3)
C4—C3—C2	120.8 (3)	C15—C16—H16	118.6
C4—C3—H3	119.6	C17—C16—H16	118.6
C2—C3—H3	119.6	C16—C17—C18	123.0 (3)
C3—C4—C5	120.9 (3)	C16—C17—C22	119.0 (3)
C3—C4—H4	119.5	C18—C17—C22	118.0 (3)
C5—C4—H4	119.5	C19—C18—C17	122.0 (3)
C6—C5—C10	119.4 (3)	C19—C18—H18	119.0
C6—C5—C4	122.4 (3)	C17—C18—H18	119.0
C10—C5—C4	118.2 (3)	C18—C19—C20	119.5 (3)
C7—C6—C5	121.9 (3)	C18—C19—H19	120.3
C7—C6—H6	119.1	C20—C19—H19	120.3
C5—C6—H6	119.1	C21—C20—O3	125.9 (3)
C6—C7—C8	117.9 (3)	C21—C20—C19	121.0 (3)
C6—C7—C12	119.6 (3)	O3—C20—C19	113.1 (3)
C8—C7—C12	122.4 (3)	C20—C21—C22	120.3 (3)
C9—C8—C7	120.8 (3)	C20—C21—H21	119.9
C9—C8—H8	119.6	C22—C21—H21	119.9
C7—C8—H8	119.6	C23—C22—C21	123.2 (3)
C8—C9—C10	121.9 (3)	C23—C22—C17	117.6 (3)
C8—C9—H9	119.0	C21—C22—C17	119.2 (3)
C10—C9—H9	119.0	C24—C23—C22	122.2 (3)
C9—C10—C11	122.8 (3)	C24—C23—H23	118.9
C9—C10—C5	118.0 (3)	C22—C23—H23	118.9
C11—C10—C5	119.1 (3)	C23—C24—C15	120.7 (3)
C2—C11—C10	121.1 (3)	C23—C24—H24	119.6
C2—C11—H11	119.4	C15—C24—H24	119.6
C10—C11—H11	119.4	O3—C25—H25A	109.5
O2A—C12—C13	118.5 (3)	O3—C25—H25B	109.5
O2A—C12—C7	120.7 (3)	H25A—C25—H25B	109.5
C13—C12—C7	120.8 (3)	O3—C25—H25C	109.5
C13—C12—H12B	119.6	H25A—C25—H25C	109.5
C7—C12—H12B	119.6	H25B—C25—H25C	109.5
C14—C13—C12	121.7 (3)		
			
C1—O1—C2—C11	−0.5 (5)	C12—C13—C14—O2B	30.1 (7)
C1—O1—C2—C3	−179.7 (3)	C12—C13—C14—C15	178.3 (3)
C11—C2—C3—C4	1.7 (5)	C13—C14—C15—C16	165.1 (3)
O1—C2—C3—C4	−179.0 (3)	O2B—C14—C15—C16	−52.0 (9)
C2—C3—C4—C5	−0.9 (5)	C13—C14—C15—C24	−16.7 (5)
C3—C4—C5—C6	178.3 (3)	O2B—C14—C15—C24	126.2 (8)
C3—C4—C5—C10	−0.4 (4)	C24—C15—C16—C17	0.1 (4)
C10—C5—C6—C7	−2.9 (4)	C14—C15—C16—C17	178.4 (3)
C4—C5—C6—C7	178.4 (3)	C15—C16—C17—C18	179.7 (3)
C5—C6—C7—C8	1.4 (4)	C15—C16—C17—C22	−1.3 (4)
C5—C6—C7—C12	178.2 (3)	C16—C17—C18—C19	178.6 (3)
C6—C7—C8—C9	0.9 (5)	C22—C17—C18—C19	−0.3 (4)
C12—C7—C8—C9	−175.8 (3)	C17—C18—C19—C20	−1.3 (5)
C7—C8—C9—C10	−1.6 (5)	C25—O3—C20—C21	−6.3 (5)
C8—C9—C10—C11	−179.9 (3)	C25—O3—C20—C19	175.6 (3)
C8—C9—C10—C5	0.0 (4)	C18—C19—C20—C21	2.4 (5)
C6—C5—C10—C9	2.2 (4)	C18—C19—C20—O3	−179.4 (3)
C4—C5—C10—C9	−179.1 (3)	O3—C20—C21—C22	−179.7 (3)
C6—C5—C10—C11	−177.9 (3)	C19—C20—C21—C22	−1.8 (5)
C4—C5—C10—C11	0.8 (4)	C20—C21—C22—C23	−179.7 (3)
O1—C2—C11—C10	179.5 (3)	C20—C21—C22—C17	0.1 (4)
C3—C2—C11—C10	−1.3 (4)	C16—C17—C22—C23	1.8 (4)
C9—C10—C11—C2	179.9 (3)	C18—C17—C22—C23	−179.2 (3)
C5—C10—C11—C2	0.0 (4)	C16—C17—C22—C21	−178.0 (3)
C6—C7—C12—O2A	−22.7 (5)	C18—C17—C22—C21	1.0 (4)
C8—C7—C12—O2A	154.0 (3)	C21—C22—C23—C24	178.7 (3)
C6—C7—C12—C13	159.5 (3)	C17—C22—C23—C24	−1.2 (4)
C8—C7—C12—C13	−23.8 (5)	C22—C23—C24—C15	0.0 (4)
O2A—C12—C13—C14	−14.0 (5)	C16—C15—C24—C23	0.6 (4)
C7—C12—C13—C14	163.8 (3)	C14—C15—C24—C23	−177.6 (3)

### Hydrogen-bond geometry (Å, º)

Cg2 and Cg4 are the centroids of rings C5-C10 and C17-C22, respectively.

**Table:**

*D*—H···*A*	*D*—H	H···*A*	*D*···*A*	*D*—H···*A*
C9—H9···*Cg*4^i^	0.93	2.86	3.543 (4)	131
C18—H18···*Cg*2^ii^	0.93	2.85	3.611 (4)	140
C23—H23···*Cg*2^iii^	0.93	2.88	3.592 (4)	134

Symmetry codes: (i) −*x*+2, *y*−1/2, −*z*+3/2; (ii) −*x*+1, *y*+1/2, −*z*+3/2; (iii) −*x*+2, −*y*+1, −*z*+2.
