# Supplementary figures and images for: Crystal structure of (E)-1,3-bis­(6-methoxy­naphthalen-2-yl)prop-2-en-1-one
Source: Acta Crystallogr E Crystallogr Commun. 2015 Oct 24;71(Pt 11):o884–5. doi: 10.1107/S2056989015019714 (PMC4644999; doi:10.1107/S2056989015019714)

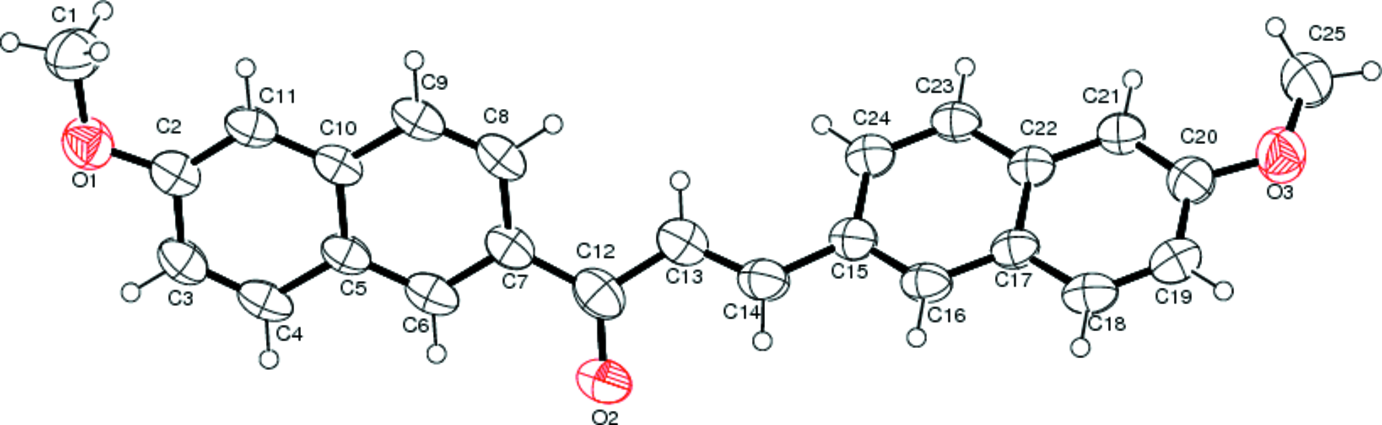

Supplement: Supplementary file 4 [file e-71-0o884-fig1.tif]

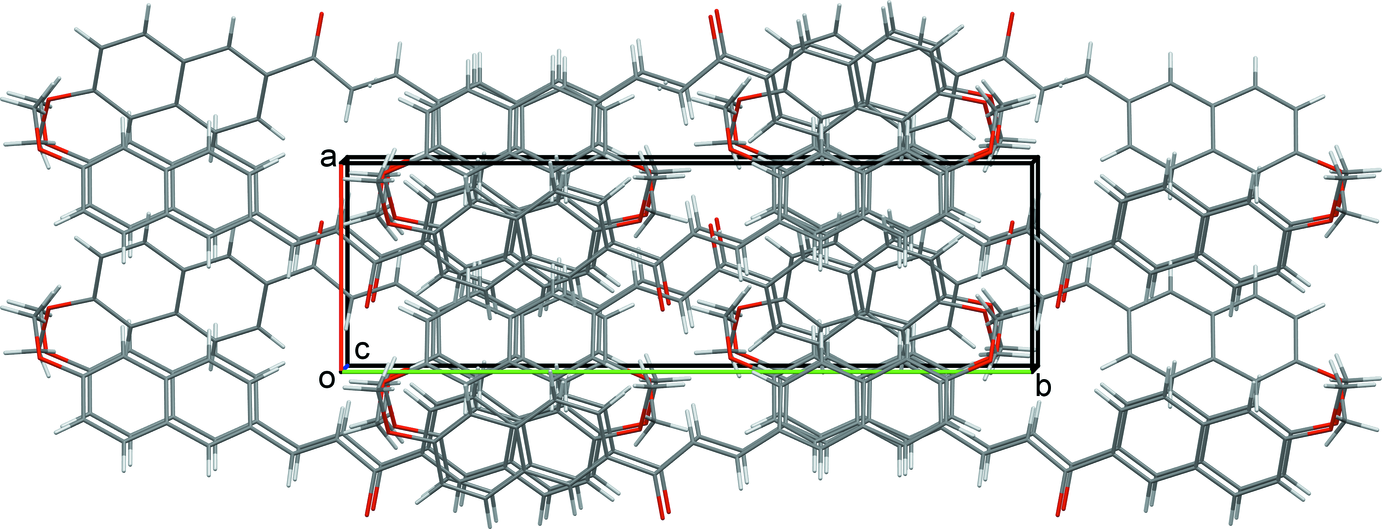

Supplement: Supplementary file 5 [file e-71-0o884-fig2.tif]
